# Randomised controlled pilot study to assess the feasibility of a Mediterranean Portfolio dietary intervention for cardiovascular risk reduction in HIV dyslipidaemia: a study protocol

**DOI:** 10.1136/bmjopen-2015-010821

**Published:** 2016-02-08

**Authors:** Clare Stradling, G Neil Thomas, Karla Hemming, Gary Frost, Isabel Garcia-Perez, Sabi Redwood, Shahrad Taheri

**Affiliations:** 1 University of Birmingham, Birmingham, UK; 2 Imperial College London, London, UK; 3 University of Bristol, Bristol, UK; 4 Clinical Research Core and Department of Medicine, Weill Cornell Medicine in Qatar and New York, Doha, Qatar

**Keywords:** NUTRITION & DIETETICS

## Abstract

**Introduction:**

HIV drug treatment has greatly improved life expectancy, but increased risk of cardiovascular disease remains, potentially due to the additional burdens of infection, inflammation and antiretroviral treatment. The Mediterranean Diet has been shown to reduce cardiovascular risk and mortality in the general population, but no evidence exists for this effect in the HIV population. This study will explore the feasibility of a randomised controlled trial (RCT) to examine whether a Mediterranean-style diet that incorporates a portfolio of cholesterol-lowering foods, reduces cardiovascular risk in people with HIV dyslipidaemia.

**Methods and analysis:**

60 adults with stable HIV infection on antiretroviral treatment and low-density lipoprotein cholesterol >3 mmol/L will be recruited from 3 West Midlands HIV services. Participants will be randomised 1:1 to 1 of 2 dietary interventions, with stratification by gender and smoking status. Participants allocated to Diet1 will receive advice to reduce saturated fat intake, and those to Diet2 on how to adopt the Mediterranean Portfolio Diet with additional cholesterol-lowering foods (nuts, stanols, soya, oats, pulses). Measurements of fasting blood lipids, body composition and arterial stiffness will be conducted at baseline, and month 6 and 12 of the intervention. Food intake will be assessed using the Mediterranean Diet Score, 3-day food diaries and metabolomic biomarkers. Questionnaires will be used to assess quality of life and process evaluation. Qualitative interviews will explore barriers and facilitators to making dietary changes, and participant views on the intervention. Qualitative data will be analysed using the Framework Method. Feasibility will be assessed in terms of trial recruitment, retention, compliance to study visits and the intervention. SD of outcomes will inform the power calculation of the definitive RCT.

**Ethics:**

The West Midlands Ethics Committee has approved this study and informed consent forms. This trial is the first to test cholesterol-lowering foods in adults with HIV.

**Trial registration number:**

ISRCTN32090191; Pre-results.

**Strengths and limitations of this study**

- This is a randomised, multicentred, dietary intervention pilot study in adult HIV centres in the UK, with subjective and objective dietary assessment.
- The outcome and process evaluation data will inform a large definitive trial.
- The feasibility study design is not powered to measure effect size.

## Introduction

Highly active antiretroviral therapy (ART) for the treatment of HIV infection has significantly improved life expectancy.^1^ However, HIV patients are increasingly experiencing metabolic complications, such as dyslipidaemia and insulin resistance, that increase cardiovascular disease (CVD), which is now the commonest cause of death in the optimally treated HIV population.^1^ The cluster of dyslipidaemia, smoking and poor-quality diets appears to drive the increased risk of CVD in the HIV population.^2^ However, when compared with case-matched controls, people with HIV infection exhibit a 50% increased risk of myocardial infarction beyond that explained by recognised risk factors.^3^ Therefore, other factors have been proposed for the excess CVD observed, including the actions of specific antiretroviral drugs and the direct impact of the HIV virus. Specific ART drugs have been observed to be associated with increased risk of myocardial infarction in a large international cohort;^4^ this effect is only partly explained by their effect on lipids.^5^ HIV infection per se potentially increases cardiovascular (CV) risk via mechanisms involving inflammation,^6^ ^7^ CD4 cell count depletion,^8^ altered coagulation,^9^ dyslipidaemia,^10^ impaired arterial elasticity^11^ and endothelial dysfunction.^12^ In summary, the risk of CVD is elevated in the HIV population via multiple mechanisms.

Dyslipidaemia is a key risk factor for CVD in those with HIV infection. The current international guidelines recommend dietary intervention as the first-line treatment for HIV dyslipidaemia.^13–15^ These guidelines are based on targeting the general population^16^ ^17^ where dietary advice, with emphasis on reduction of saturated fat, has been shown to reduce CVD risk and mortality.^18^ Presuming that the reduction in CVD risk achieved by dietary intervention in the general population will be mirrored in the HIV population is problematic for two reasons. First, because the precise underlying mechanism of the increased risk of CVD in HIV is unclear^19^ and there is a need to consider multiple factors such as infection, inflammation and antiretroviral treatment. Second, there is debate over the focus on saturated fat reduction in the absence of consideration of the source of the saturated fat (vegetable or animal fats) and the nature of the carbohydrates it displaces.

Given the uncertainties regarding the impact of dietary interventions on CVD in the HIV population, this study set out to clarify the potential of the dietary intervention on modifying low-density lipoprotein cholesterol (LDL-cholesterol), a major marker of increased CVD risk, in the HIV population.

### Rationale for intervention

#### Saturated fat and the National Cholesterol Education Program

Our meta-analysis of pooled results from four randomised controlled trials (RCTs) demonstrated a clinical benefit of dietary intervention in reducing triglycerides by −0.46 mmol/L (95% CI −0.85 to −0.07 mmol/L) over an average treatment period of 8 months in HIV patients on antiretroviral therapy, but reported no effects on LDL-cholesterol or high-density lipoprotein cholesterol (HDL-cholesterol).^20^ That review clearly identified the need for well-designed, robust trials. One subsequent trial suggested an improvement in HDL-cholesterol and highly sensitive C reactive protein (a marker of CVD risk) as secondary outcomes, although it was likely underpowered to assess the effects of lifestyle modification on these.^21^ However, the lack of effect on LDL-cholesterol may indicate that the interventions used (National Cholesterol Education Program (NCEP) Therapeutic Lifestyle Changes diet emphasising reduction in saturated fat to 7% of total calories) are insufficient to independently alter HIV dyslipidaemia and associated CV risk.

#### Mediterranean Diet

The Mediterranean Diet is characterised by: high consumption of olive oil, legumes, unrefined cereals, fruits and vegetables, and moderate consumption of dairy products, fish and wine, but low consumption of meat. The Mediterranean Diet has been examined in HIV cohort studies, with reports of no association between adherence to the Mediterranean Diet and plasma lipid changes during the first year of ART treatment.^22^ Favourable associations have been reported with insulin resistance and HDL-cholesterol in patients on ART, but only in those with fat redistribution, such as visceral fat accumulation and/or subcutaneous fat wasting.^23^ One parallel, randomised trial has been conducted in the HIV population, comparing low-fat NCEP diet versus the same with additional Mediterranean components. The findings were unfavourable in both arms, with increases in triglyceride levels in the NCEP group and increases in cholesterol levels in the modified Mediterranean group. It was, however, a pilot study, and variance was not reported, making interpretation difficult.^24^

Despite the lack of available evidence in the HIV population, the strength of the effects of the Mediterranean Diet documented in the non-HIV population cannot be ignored. Its importance is evident from observational cohort studies,^25^ a secondary prevention trial,^26^ and a primary prevention trial^27^ demonstrating substantial reduction in CV morbidity and mortality. In a Spanish population at high CV risk, but with no disease present, the Mediterranean Diet reduced the incidence of major CV events by 30% (HR 0.70, 95% CI 0.55 to 0.89).^27^ Despite the large benefit seen in clinical end points, the effect on blood lipids was less dramatic, with no significant difference in LDL-cholesterol between the Mediterranean Diet and Low Fat Diet groups due to similar reductions of 0.15 mmol/L. The Mediterranean Diet appears to benefit HDL-cholesterol, with increased levels of 0.08 mmol/L (95% CI 0.04 to 0.10) producing a reduction in total cholesterol to HDL ratio.^28^ One UK study has explored the feasibility of implementing the Mediterranean Diet in a non-Mediterranean setting, demonstrating that Northern European CVD patients could adopt and maintain a Mediterranean Diet.^29^

#### Functional foods and the Portfolio Diet

American guidelines have recommended the use of functional foods as additional options to enhance the effectiveness of cholesterol-lowering diets.

Foods with cholesterol-lowering properties have been examined in clinical trials in the general population, demonstrating 6% reductions in LDL-cholesterol with 15 g/day soya protein;^30^ 7–10% reductions with 2 g/day plant stanols;^31^ 3–5% reductions with 3 g/day β-glucans in oats;^32^ and a dose-dependent effect of 1% LDL-cholesterol reduction with every 4–11 g of nut consumption.^33^ When these cholesterol-lowering foods (plant sterols, soy protein, viscous fibre and almonds) were combined into a ‘Portfolio Diet’, the effect was magnified, producing an LDL-cholesterol reduction of −1.36 mmol/L (29%) in short-term feeding trials.^34^ Reductions of 29% were sustained at 1 year in a free-living trial, under real-life conditions, but only for the one-third of the participants who achieved complete adherence. The treatment effect was halved to 13% LDL-cholesterol reduction (−0.61 mmol/L)^35^ in the intention-to-treat analysis, but was still impressive. This finding was consistent with LDL-cholesterol reductions of 14% (−0.67 mmol/L) in another free-living trial of 6 months, where adherence to the portfolio components was only 46%.^36^

### Motivational interviewing

Dietary interventions are a form of behavioural intervention to help improve adoption of behaviour change. The UK National Institute for Health and Care Excellence (NICE) guidelines (2007) recommend selecting interventions that motivate and support people to feel positive about the benefits of health-enhancing behaviours.^37^ These recommendations apply to motivational interviewing, which has been shown to be effective in the domain of diet and exercise.^38^ Motivational interviewing was developed by Miller and Rollnick, and subsequently ascribed to the transtheoretical model of change.^39^ Motivational interviewing is based on four key principles: expressing empathy, developing discrepancy, rolling with resistance and enhancing self-confidence. Systematic reviews in obesity management,^40^ diabetes prevention^41^ and CV risk reduction in high-risk groups,^42^ indicate that such robust behavioural change strategies are key to effective lifestyle change programmes. Guidelines for diabetes prevention^43^ and obesity prevention/management^44^ recommend individual level intervention using established, well-defined behaviour change techniques (goal setting, relapse prevention, self-monitoring, motivation interviewing, prompting self-talk, individual tailoring and time management). In this study, motivational interviewing will facilitate the development of individualised approaches to support behaviour change in relation to diet, enhance participant self-efficacy and motivate participants to change, achieve their goals and maintain behaviours.

### Intervention proposed for this trial

The hypothesis of the current study is to combine the effect observed in the general population of the CV event reduction from the Mediterranean Diet with the cholesterol reduction from the functional foods of the Portfolio Diet. This dietary advice is to be delivered in the motivational interviewing style, as advocated by HEART UK in their Ultimate Cholesterol Lowering Plan (UCLP), which is also based on the Portfolio Diet. Therefore, this study aims to examine whether a diet that is low in saturated fat, and incorporates cholesterol-lowering foods and a Mediterranean Diet style, reduces blood lipid levels in patients with stable HIV infection on antiretroviral treatment.

The study is not designed to determine which of the food or nutrient components are responsible for the effect observed, but to assess the combined impact of the two dietary patterns (low saturated fat and Mediterranean Portfolio Diet) on LDL-cholesterol. The reason for investigating dietary patterns rather than examining isolated nutrients is that they are more relevant to the ways people choose their foods and the possibility of observing the potential additive or synergistic effects of combining different dietary components.^45^

A parallel pilot trial design with an active comparator was selected for two reasons. First to ensure that entering the study would not disadvantage participants who had access to dietary advice as part of their current standard of care. Second, to prevent performance bias resulting from differential levels of attention between groups, and the preferred outcomes of the potential participants.

The 2008 Medical Research Council Framework^46^ was followed for the development of this complex intervention, including examining the theoretical basis, developing the intervention with patient input, using patient focus groups to identify potential barriers to recruitment, and noting preferred outcomes. Based on the evidence from this development process thus far, a pilot trial is proposed to test the feasibility and acceptability of procedures for recruitment, allocation, retention and intervention, to inform the viability of conducting a definitive multicentre trial to evaluate the effectiveness of this specific dietary intervention in this population.

### Study objectives

The aim of this study is to assess the feasibility, and pilot the design and delivery of a RCT that will subsequently form the framework for a definitive RCT to evaluate the effectiveness of dietary intervention on CV risk of people with HIV dyslipidaemia. The objectives are:

1. To obtain reliable estimates for recruitment, retention and study attendance compliance;
2. To explore the appropriateness of the trial procedures, design and duration;
3. To understand how the intervention works in practice and its acceptability from the participants’ perspective;
4. To quantify levels of adherence to the dietary intervention and identify contextual factors associated with variation;
5. To conduct process evaluation to assess fidelity and quality of implementation;
6. To determine estimates of the variability of the outcome measures and markers of CV risk, anthropometric measures and quality of life, including LDL-cholesterol, pulse wave velocity and waist circumference.

## Methods and analysis

### Design

This is a two-arm, parallel, multicentre RCT for people with HIV dyslipidaemia. The two arms are:

1. Diet1—reduced saturated fat;
2. Diet2—Mediterranean Portfolio Diet.

**Figure 1:**
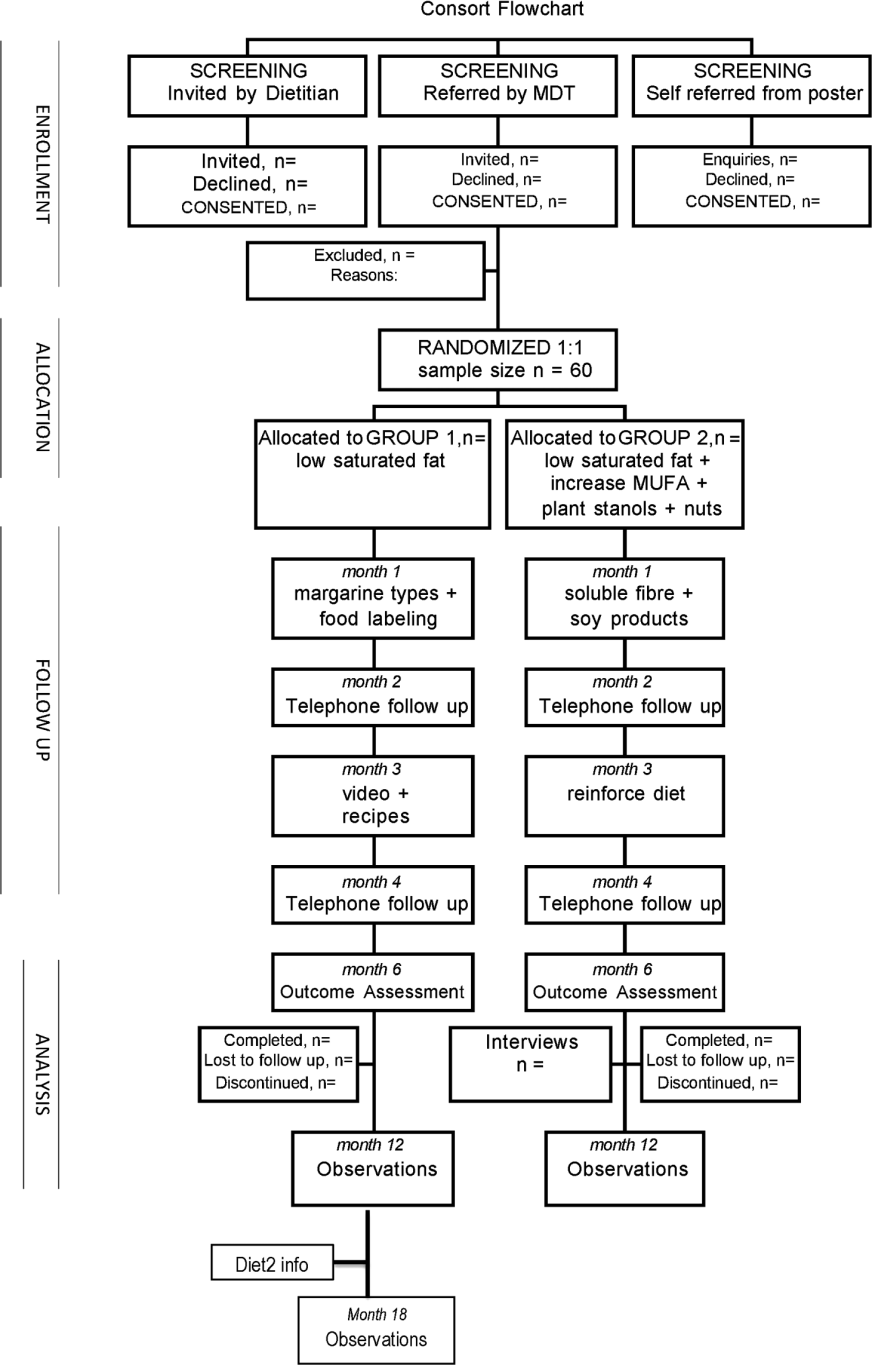
Consort  flow diagram for Best Foods for Your Heart trial. MDT, multi-disciplinary team; MUFA, monounsaturated fatty acids.

Randomisation will be stratified according to gender and smoking status. The trial is funded by the National Institute for Health Research (NIHR) Doctoral Fellowship Programme and has been registered with the International Standard Randomised Controlled Trial Number registry (identifier: ISRCTN32090191). The study flow chart in figure 1 shows progression through the study for individual participants.

### Setting

The study is a multicentre study set in three HIV services in the West Midlands (Birmingham Heartlands Hospital, Queen Elizabeth Hospital Birmingham and City of Coventry Health Centre). The West Midlands represents 10% of the population of England and Wales, and spans the range of population densities, urbanisation and socioeconomic profiling typical of the UK. It has the largest ethnic diversity outside of London, with 17.3% of the population being African, Caribbean or Asian.^47^

### Participants

The target population is adults attending HIV services within the West Midlands who have been stable on ART treatment for >6 months, and who have viral suppression and LDL-cholesterol >3 mmol/L. The inclusion criteria are: fluency in English and intent to stay in the UK for 1 year. The exclusion criteria are: planning pregnancy in next 6 months; current use of lipid-lowering agents (any interfering drug or diet); secondary causes of dyslipidaemia (renal or liver disease, diabetes, hypothyroidism, familial hyperlipidaemia); known nut allergy; unstable psychiatric disorder (including eating disorders); current participation in a weight loss programme or other dietary intervention; and inability to understand printed materials. When in doubt, the opinion and approval of the trial investigation team will be sought.

### Sample size

A CI approach was used to estimate the sample size required to establish feasibility.^48^ The criterion for success is that the definitive trial will be feasible if the attrition rate is <20% (or complete follow-up in at least 80% of all recruited participants). With a sample size of 60 participants, the expected attrition rate of 20% can be estimated with a 20% (95% CI 10% to 30%) margin of error. This upper bound of 30% is appropriate as it is the cut-off used to determine which trials constitute best evidence for behavioural intervention^49^ and which trials qualify to receive funding for implementation by the Centers for Disease Control and Prevention 2010.^50^

### Recruitment

Recruitment will be conducted using a number of methods: advertising poster in waiting room for patients; referral from multidisciplinary team providing HIV care; and screening of patient blood results for LDL-cholesterol >3 mmol/L. Patients will be assessed for eligibility by the researcher. Eligible patients will be handed or posted an invitation letter and participant information sheet containing details of all the interventions and assessments to be expected. To avoid contamination, the description of the dietary interventions will be general and applicable to both groups. Patients will be given at least 24 h to consider the information before being contacted to confirm the date of their next routine blood test appointment, and reminded to attend in a fasted state, with no food consumed 12 h prior to the appointment time. Written informed consent will be obtained from all participants at their first trial visit, prior to baseline measurements.

### Allocation strategy

Participants will be randomly allocated to Diet1 or Diet2 in a 1:1 ratio on their second trial visit. A statistician from the University of Birmingham will produce a computer-generated allocation sequence using random block sizes of 2 and 4, stratified by gender and smoking status. Block sizes will be concealed until completion of the trial. The research dietitian will allocate participants according to the diet number concealed in the next sequentially numbered, opaque, sealed envelope, relevant for their gender and smoking status. As this is a complex intervention, it is not possible to blind the participants, nor is it possible to blind the healthcare professionals. The terms Diet1 and Diet2 will be used with the aim of achieving participant blinding to the exact content of the diet and type of foods included, to prevent internet searching of diet titles and potential contamination between groups. The success of the level of blinding achieved will be evaluated using questions on intervention preference and self-belief in success.

### Data to be collected at baseline, month 6 and 12

Sociodemographic data on age, gender, ethnicity and occupational status will be collected. Biomedical data will be collected on weight, height, body mass index, waist circumference, body composition and stool frequency/bowel habit. Weight and body composition will be measured in light clothing, barefoot, on a Class 3 Tanita BC420SMA weighing scale (meets Medical Device Directive and Non-Automatic Weighing Instruments European regulations) (Tanita, Tokyo, Japan). Participants will be asked to abstain from food and drink for 4 h prior to the assessment. Height will be measured to 0.1 cm using stadiometers with the supported stretch stature method. Waist circumference will be measured horizontally at the top of the iliac crest, with a flexible non-stretching Seca tape, to 0.1 cm. Bowel habit will be assessed via self-report, to ascertain any disturbances that may require fibre and fluid intake modification.

Lifestyle data will be collected on smoking status, alcohol intake and CV risk factors, to enable calculation of QRISK 10-year CV risk.^51^ Blood pressure will be measured seated, with automated sphygmomanometer (Omron, The Netherlands), taking the average of three consecutive readings. Intensity of physical activity will be measured objectively using the Actigraph GT3X+ accelerometer (Actigraph, Florida, USA), a triaxial movement sensor that also records step counts. It will be worn over the right hip for 7 days, during waking hours, as this is the ideal period to obtain consistent measures.^52^ Mean counts per minute will be compared between groups, to identify any potential influence of physical activity as a confounding factor. Best practices will be followed with regard to monitor use protocols, calibration and analysis of accelerometer data.^53^

Aortic pulse wave velocity, as a measure of arterial stiffness, is an independent predictor of CV risk and mortality.^54^ Aortic pulse wave velocity will be measured from the right carotid and femoral arteries with the Vicorder system (Skidmore Medical, UK).^55^ Duplicate recordings will be made and saved, once a steady, consistent pattern of pulse waveforms is achieved and maintained for at least 10 cardiac cycles.

Blood samples will be collected and stored for later measurement of apolipoprotein A and B, inflammatory markers (highly sensitive C reactive protein) and insulin sensitivity (fasting insulin, adiponectin). Vitamin E levels will be measured to monitor any potential effect of plant stanols on absorption of fat-soluble vitamins.

#### Dietary assessment

A number of different dietary assessment tools will be used to enable cross-validation of methods and exploration of a variety of different strategies for selective implementation in future trials. Adherence to a Mediterranean-style diet will be assessed using a 14-item tool used in the PREDIMED study.^27^ ^56^ Fruit and vegetable intake will be assessed using a two-item questionnaire, previously used in studies on healthy adults from diverse ethnic backgrounds living in a low-income neighbourhood^57^ and patients with CVD,^58^ and validated against plasma and urine biomarkers. A compliance score will assess consumption of the functional foods components of the Mediterranean Portfolio Diet intervention at month 6 and 12.^35^ Dietary intake will be assessed using a 3-day food diary, recorded either on paper or on the mobile phone app MyNetDiary (MyNetDiary Inc, New Jersey, USA). Kitchen scales will be provided to assess food quantities. The researcher will review the diaries with participants to clarify brands, methods of food preparation and recall of missing foods.^59^ Where weights of foods are unknown, they will be estimated using information from Ministry of Agriculture, Fisheries and Food Portion Sizes.^60^ Nutritional analysis will be conducted using DietPlan7 dietary analysis software (Forestfield Software Limited, Horsham). Metabolomic biomarkers will be used to independently assess dietary compliance, including trimethylamine-N-oxide and 1-methylhistidine for oily fish, urolithin A glucuronide for nut intake, equol for soy isoflavone, and linoleate, oleate and palmitate for olive oil intake. Urine sample from 24 h collection at baseline and month 6 will be stored for later metabolic profiling analysis by mass spectrometry using an analytical platform based on ^1^H proton nuclear magnetic resonance spectroscopy and mass spectroscopy, at Imperial College London.^61^ The quantified metabolite concentrations will be individually and collectively regressed against biometric data and against the global metabolic profiles.

#### Process Evaluation

Process Evaluation questionnaires will be used at baseline and month 6. Various approaches were selected and combined in the questionnaires to incorporate the components recommended by the Medical Research Council guidelines.^46^ Fidelity and quality of implementation of the intervention will be assessed using open-response questions, to get a general overview of acceptability, as previously demonstrated in the WATCH IT evaluation,^62^ and specific Likert-ranked questions, to examine participant perceptions of the strengths and weaknesses of the programme, as used in the SHED-IT evaluation.^63^ Assessment of diet intrusiveness, the participants’ intention to make changes and self-belief in success, will be used to support clarification of causal mechanisms. Contextual factors associated with variation in outcomes will be identified via a validated measure of self-efficacy for fat intake behaviour, containing three task-specific domains of self-efficacy (negative mood, positive mood and food availability), which have been shown to predict different types of fat reduction behaviours in low-income populations, with variations according to race.^64^ In trials, such as this one, where blinding is not possible, the patient's preference for treatment can influence the outcome, especially where the participant is not simply a passive recipient of the intervention, but is required to engage and make lifestyle changes. Therefore, intervention group preferences will be elicited before each randomisation, and used subsequently in the analysis of covariance (ANCOVA) to investigate the influence on LDL-cholesterol of participants’ preferences at baseline.^65^

#### Quality of life

One generic (EQ-5D) and one HIV-specific (MOS-HIV 35-item instrument) will be used to assess the impact of the trial on the participant's quality of life. These tools are deemed the most appropriate adjunct and HIV-targeted measures for use in HIV clinical trials.^66^ As responses to the EQ-5D thermometer tend to reflect physical more than mental health, the Warwick-Edinburgh Mental Well-being Scale (WEMWBS) will be used to measure mental well-being. The WEMWBS, a 14-item scale covering subjective well-being and psychological functioning, has been validated in the UK adult population.^67^ Permission has been granted to use these questionnaires in this study.

#### Interviews

Qualitative data will be collected from a sample of participants at the end of the 6-month intervention period. Participants will be purposively selected from the Diet2 arm to include men and women of different ethnicities, with high and low levels of adherence, to reflect the diversity and breadth of experiences of a wide range of participants. Semistructured interviews will be conducted using open-ended, non-directive questions. The aim will be to understand participants’ experience of implementing dietary changes by enabling them to ‘tell their story’—what they did and how—as well as identifying barriers to, and enablers of, success in making and maintaining diet changes. It is anticipated that there will be variations in the effectiveness of the dietary intervention for different individuals in different sociocultural settings. Analysis of the interview data will serve to describe and potentially explain these differences and the factors involved. By exploring participant views on the intervention, study design and delivery, the interviews will identify problems at the feasibility stage to prevent them occurring at the full trial stage.

Written informed consent for digital recording of the 30–60 min interview will be sought prior to booking a mutually agreed time and place, enabling the interview to be conducted either in participants’ homes or a clinic room with privacy. Participants will be encouraged to invite their partner, family member or other cohabitant to join in the interview and give their perspective on how dietary changes are lived.

The sample size will be around 10 although the actual number recruited will be determined at the point at which the researcher is satisfied that a good understanding of the barriers and facilitators to making dietary changes has been achieved.

Interviews will be transcribed verbatim. Data analysis will be conducted following the Framework Method: familiarisation, identifying a thematic framework, indexing, charting, mapping and interpretation.^68^ ^69^ Data analysis and management will be supported by NVivo10 software (QSR International). A subset of interviews will be independently coded to verify interpretation. Where possible, data triangulation with cohabitants will be used to enhance plausibility, trustworthiness and transferability of the data analysis process. Reflective notes will be taken in a field note diary immediately after each interview and throughout data analysis, to maintain reflexivity of the researcher.

### Intervention design

All participants, in both groups, will be invited to attend three individual consultations with the research dietitian, and will receive further telephone reinforcement and support during the 6-month intervention period. Appointment times will be offered between 08:00 and 19:00, enabling flexibility for participants who are working. This will be followed by a 6-month maintenance period, with routine clinic visits only. The same research dietitian, experienced in HIV nutritional care, will provide all consultations.

#### Diet1: low saturated fat

Consultations will focus on reduction of saturated fat to <10% of energy intake, in line with UK guidelines^15^ ^16^ and evidence on reducing the risk of CV events.^70^ Resources will be provided, such as written information, recipes and online videos, covering various topics including sources of saturated fat, food swaps, food labelling, cooking methods, cheese facts and margarine types.

On completion of the 12-month outcome measurements, participants in group 1 will receive the dietary information from Diet2 (Mediterranean Portfolio). Should they choose to adopt the diet, they will be given the option of having their fasting measurements repeated at month 18, at their routine blood test appointment.

#### Diet2: Mediterranean Portfolio

In addition to the information provided to group one, participants allocated to Diet2 will receive advice and support to adopt the Mediterranean Diet supplemented by additional functional foods with cholesterol-lowering properties. This will be embedded within a motivational interviewing style consultation to include assessing readiness to change, utilising decisional balance, reflective listening and open-ended questions, to identify needs, motivators and barriers to changing their diet, helping the participant to develop and verbalise arguments for change (desire, ability, reason need and commitment), and diminish resistance to it. The aim is to help empower and motivate the patient to make the changes identified during goal setting for their specific change plan. The dietitian will use the food diary to guide the participant towards the dietary changes most pertinent to their current intake while considering socioeconomic factors and family dynamics. The diet is not prescriptive, as goals will be negotiated individually with each participant during their first session and reviewed at each visit.

On the randomisation visit, daily consumption of 57 g tree nuts and 2 g plant stanols will be encouraged in the form of two handfuls of unsalted mixed nuts (almonds, cashew nuts, peanuts, Brazil nuts, hazelnuts, pecans, walnuts, pistachios, macadamia nuts) and a 50 mL cholesterol-lowering drink. At subsequent sessions, participants will be encouraged to continue with the nuts and stanols, while also aiming to eat 15 g/day soy protein as soya milk, yogurt or dessert, tofu and meat substitutes, and adopt a Mediterranean-style diet, with more vegetables and fruit, olive oil, and approximately 15–20 g/day soluble fibre from oats, pearl barley, lentils, beans and flaxseed. Supplies of the functional foods (nuts, soy protein, plant stanols, oats and pulses) will be given to participants to offset the additional cost of making dietary changes and inconvenience of searching for unusual items in supermarkets.

It is acknowledged that trials requiring longer term dietary change face challenges regarding attrition and adherence to study requirements, therefore some flexibility with dietary requirements will be provided in order to facilitate positive experiences and encourage commitment to the long term.^71^ If the participant is unable to consume the full amount of one component, for example, due to taste, they will be encouraged to maximise the contribution of other components.

In keeping with maintaining the integrity of a complex intervention, this trial will aim to standardise the function and process of the intervention, not the components themselves,^72^ thus allowing context-level adaptation, for example, use of ethnicity-appropriate resources. A sample of consultations will be audio recorded, for subsequent assessment by an HIV clinical psychologist, to monitor the quality of the motivational interviewing and fidelity of the intervention.

### Outcome measures

The primary outcomes for this study are feasibility and acceptability of trial procedures for recruitment, retention, data completion and the intervention. The protocol will be considered viable for a definitive RCT without modification if the following outcomes are met:

1. Recruitment rates of at least 50% of eligible patients;
2. Attrition rate of <20% by 6 months;
3. Compliance rate of 60% to trial.

If these outcomes are not met, the protocol will be modified in light of the study's findings.

Recruitment rate will be measured as proportion of eligible patients who are subsequently enrolled. Methods of recruitment will be compared: difference between recruitment rate from systematic methods (screening results) and ad hoc methods (posters, referral by multidisciplinary team). Reasons will be sought for declined participation. Appropriateness of eligibility criteria and their practical application for definitive trial will be explored.

Attrition rate is defined as both discontinuation and loss to follow-up of participants at 6 months. Logistic regression will be conducted to assess whether completion is associated with age, gender or allocation preference. Reasons for discontinuation will be sought from participants. The target of 20% (with precision of 10%) is based on the mean attrition rate from five Portfolio Diet studies (13%) and systematic review evidence (18%),^20^ although attrition has ranged from 25%^24^ to 42%^73^ in other HIV diet studies. Acceptability of the intervention and group allocation will be explored by questionnaire, and assessed by comparing attrition rates between the two groups.

Attendance to appointments, collection of supplied foods and completion of food diaries will be used as indicators of participation and study compliance.

### Statistical analysis plan

Descriptive findings will be explored regarding numbers recruited, completing, dropping out and summary characteristics of each arm at baseline. CI estimation, not p value, will be used as the appropriate analysis for pilot studies.^74^ For all binary outcomes, we will therefore compute the number and proportion in each arm, and 95% CI.

Exploratory analysis of the treatment effect will be conducted. Intention-to-treat analyses will be conducted with participants as originally allocated at randomisation to avoid bias. Significance levels will be set at <0.05 with two-tailed tests. Effect size for the performance outcomes will be explored using ANCOVA with adjustment for baseline value,^75^ and we will report mean differences in outcomes between arms with 95% CIs. SD of each outcome will be estimated, to inform the power calculation for a definitive trial.

Further exploratory analysis will be piloted to highlight any potential modifications required for the definitive trial. This will include integrating process and outcome data to maximise the interpretation of results.^76^ For example, to address the question of the relationship between LDL-cholesterol and variation in adherence levels to the diet, on-treatment analysis will be used, in which results for participants who adhered to the diet will be compared with results from those allocated to the diet (standard intention-to-treat approach). To address the question regarding differences in responses to the intervention between subgroups of participants, regression analysis will be used in the definitive trial (as the power will be insufficient in the pilot study), with tests for interactions to identify participants most and least likely to benefit from the intervention.

Although this trial is not sufficiently powered to compare outcome between intervention arms, estimates of differences between arms will be compared. In the event that the effect of the intervention is large (in the region of 0.5 mmol/L or 10% difference in LDL-cholesterol reduction between groups at 6 months) this study will have sufficient power and it will be important not to miss such large effect sizes if they exist.

## Dissemination

The Trial Steering Committee will provide overall trial supervision. The main ethical consideration is to ensure that the risk of harm to participants is minimised and that they are fully informed of any risks. Lipid lowering agents are not permitted during the trial. Should an individual's LDL-cholesterol rise above >5 mmol/L, this will be recorded as an adverse event and they will be withdrawn from the study, with general practitioner referral for appropriate medication.

The research dietitian, at study visits, will monitor the condition of the participants during the trial, requesting evaluation of potential harms by the participants’ HIV physician during routine clinic visits. All adverse events, whether considered trial-related or not, will be documented in the participant's case report form, and reported to the Trial Steering Committee and Sponsor.

Participants will be free to withdraw from the study at any time. If participants withdraw from the intervention, they will be asked if they would allow data to be collected at routine clinic appointments.

Electronic trial data will be entered into the encrypted and password protected trial computer, stored on the secure hospital server and archived for 5 years after study completion. Paper copies will be held in the research office for purposes of potential data checking, and shredded 1 year after study completion.

## Discussion

This study is timely for several reasons. First, CVD remains the largest cause of mortality in the UK^77^ and will continue to rise in the ageing HIV population. Second, current guidelines recommend dietary intervention as first-line treatment for HIV dyslipidaemia.^13–15^ These recommendations are based on guidelines for the general population^16^ ^17^ where dietary advice, with emphasis on reduction of saturated fat, has been shown to reduce CVD risk and mortality.^18^ Presuming that the reduction in CVD achieved by dietary intervention in the general population will be mirrored in the HIV population is problematic, given the complexity of CVD among the HIV population and the potential of different contributing mechanisms.^19^ Third, there are no studies on dietary intervention, of any kind, in the HIV population in the UK. Also, there are no known studies on cholesterol-lowering foods, nor on the Portfolio Diet, in the HIV population internationally. Components of this diet have been incorporated into the UCLP (HEART UK), but have yet to be evaluated in the UK population. Therefore, findings from this feasibility study utilising the Mediterranean Portfolio Diet in the HIV population could potentially inform the acceptability of this diet in the wider population.

As indicated by the lack of effect found with diets low in saturated fat, further studies with interventions of greater intensity and sufficient duration are required to elucidate the full potential of dietary intervention, and to ascertain whether modifying the known marker of increased risk (LDL-cholesterol) modifies the risk itself (CVD). Therefore, this study aims to examine whether a diet low in saturated fat, which also incorporates functional foods and a Mediterranean style, reduces blood lipid levels in patients with HIV infection. If this dietary intervention can significantly reduce blood lipid levels, patients may have the option of reducing their CV risk without the need for lipid-lowering medications (with attendant increasing pill and side effects) or switching antiretroviral drugs (with the potential risk of losing virological control of HIV). A reduction of 1 mmol/L in LDL-cholesterol reduces the risk of dying from heart disease by nearly 20% in the general population,^78^ but this has not been ascertained in the HIV population.

Piloting this trial format will be important in determining any feasibility challenges and will be used to estimate the time, resources and sample size required for a full-scale RCT to answer these questions.
